# Post-Training Sleep Modulates Topographical Relearning-Dependent Resting State Activity

**DOI:** 10.3390/brainsci11040476

**Published:** 2021-04-09

**Authors:** Michele Deantoni, Thomas Villemonteix, Evelyne Balteau, Christina Schmidt, Philippe Peigneux

**Affiliations:** 1 Neuropsychology and Functional Neuroimaging Research Unit (UR2NF) at CRCN—Centre for Research in Cognition and Neurosciences and UNI—ULB Neurosciences Institute, Université Libre de Bruxelles (ULB), CP191 Av. F. Roosevelt 50, 1050 Bruxelles, Belgium; michele.deantoni@uliege.be (M.D.); t.villemonteix@gmail.com (T.V.); 2 CRC-GIGA In Vivo Imaging, Université de Liège, Allée du 6 Août, Bâtiment B30, Sart Tilman, 4000 Liège, Belgium; e.balteau@uliege.be (E.B.); christina.schmidt@uliege.be (C.S.); 3 Psychopathology and Neuropsychology Lab, Paris 8 University, Rue de la Liberté 2, 93,526 Saint-Denis, France; 4 Psychology and Neurosciences of Cognition (PsyNCog), Université de Liège, Quartier Agora, Place des Orateurs, 3, Bâtiment B33, 4000 Liège, Belgium

**Keywords:** functional MRI, sleep deprivation, memory consolidation, resting-state fMRI, ALFF, spatial learning

## Abstract

Continuation of experience-dependent neural activity during offline sleep and wakefulness episodes is a critical component of memory consolidation. Using functional magnetic resonance imaging (fMRI), offline consolidation effects have been evidenced probing behavioural and neurophysiological changes during memory retrieval, i.e., in the context of task practice. Resting state fMRI (rsfMRI) further allows investigating the offline evolution of recently learned information without the confounds of online task-related effects. We used rsfMRI to investigate sleep-related changes in seed-based resting functional connectivity (FC) and amplitude of low frequency fluctuations (ALFF) after spatial navigation learning and relearning. On Day 1, offline resting state activity was measured immediately before and after topographical learning in a virtual town. On Day 4, it was measured again before and after relearning in an extended version of the town. Navigation-related activity was also recorded during target retrieval, i.e., online. Participants spent the first post-training night under regular sleep (RS) or sleep deprivation (SD) conditions. Results evidence FC and ALFF changes in task-related neural networks, indicating the continuation of navigation-related activity in the resting state. Although post-training sleep did not modulate behavioural performance, connectivity analyses evidenced increased FC after post-training SD between navigation-related brain structures during relearning in the extended environment. These results suggest that memory traces were less efficiently consolidated after post-learning SD, eventually resulting in the use of compensatory brain resources to link previously stored spatial elements with the newly presented information.

## 1. Introduction

Evidence ranging from cell recordings in rodents to human behavioural and neuroimaging data show that sleep participates in the long-term consolidation of recently acquired information [1] in various memory domains [2,3]. Amongst others, sleep supports the consolidation of spatial memory [4,5,6], the cognitive system responsible for recording information about one’s environment and spatial orientation. Spatial and episodic declarative memory share similar neuroanatomical foundations in human beings and animals [7]. This makes spatial memory an attractive paradigm to study the effects of sleep, with unique opportunities for translational inferences.

Non-invasive neuroimaging studies have consistently shown that spatial navigation is subtended by a distributed brain network encompassing the hippocampus, the dorsal striatum, the precuneus and the entorhinal, parahippocampal, retrosplenial and frontal cortices [8,9]. Rodent studies further demonstrated that the hippocampus is involved in the rapid acquisition of spatial information at an early stage of learning, allowing the animal to reach its target from any starting position [10]. The entorhinal cortex contributes to the encoding of map-like spatial codes, whereas parahippocampal and retrosplenial cortices provide significant inputs allowing to anchor these cognitive maps to fixed environmental landmarks. Spatial encoding- and retrieval-related activity in these brain areas is coordinated with frontal lobe activity to plan routes during navigation [9]. Besides, navigation performance also relies on the dorsal striatum, which supports a complementary learning system based on rewarded stimulus-response automated behavioural associations, such as gradually learning successive body turns in response to environmental cues to reach a known target location from a starting point [10,11]. Human and animal studies investigating the role of hippocampal and striatal areas in place-based vs. response-based navigation strategies suggest that these two systems initially compete with each other during the acquisition phase, and then become more integrated and interdependent after extended training [12,13].

The reinstatement of experience-dependent neural activity during sleep is thought to be a critical element of memory consolidation, allowing new, fragile memory traces to stabilize and reorganize over time [14]. Supporting this hypothesis, rodent studies showed that learning-related neural patterns of hippocampal place cell activity are spontaneously replayed during sleep, in particular during non-rapid eye movement (NREM) sleep [15,16], which does not preclude an important role for REM sleep in memory consolidation (see e.g., [17,18]). Likewise, human studies evidenced the experience-dependent reactivation of neural activity during post-training sleep in brain areas previously activated at learning during wakefulness [4,19,20,21,22]. Regarding spatial memories, positron emission tomography highlighted increased activity during NREM sleep in navigation-related hippocampal and parahippocampal regions in participants trained to explore a virtual city before sleep, as compared to subjects trained to another, non-hippocampus-dependent procedural memory task [4]. Moreover, overnight gains in navigation performance were correlated with the amplitude of hippocampal activity during NREM sleep. Besides, functional magnetic resonance imaging (MRI) and magnetoencephalography investigations showed that post-learning sleep (as compared to the same time spent awake) leads to the reorganization and/or optimization of the brain patterns underlying the delayed retrieval of topographical [5,23], declarative [24] or motor procedural [25,26,27,28] memories. In particular, it was found using a spatial navigation task that post-learning sleep leads to a restructuration of the neuronal underpinnings of behaviour [5]. Navigation in a recently learned environment, initially subtended by hippocampus-based spatial strategies, becomes more contingent on response-based strategies mediated by the striatum a few days later when participants are allowed to sleep on the first night after learning [5,23], suggesting that post-learning sleep contributes to the automation of the navigation behaviour.

While providing us with relevant knowledge regarding the brain networks supporting cognitive functioning during a learning session or at delayed retrieval, online task-based functional MRI (fMRI) only indirectly explores the offline consolidation processes subtending the maintenance and optimization of novel information outside of the learning and/or testing environments, as it does not directly measure brain activity during the offline consolidation phase. We previously showed using fMRI that novel topographical learning actually modulates hippocampal responses at wake during a subsequent, unrelated attentional task, suggesting that modulated brain activity during post-training wakefulness contributes to memory consolidation by shaping and reinforcing the functional connections between learning-related cerebral structures and other brain regions [29]. Another experimental approach to investigate the offline evolution of the cerebral correlates of recently formed memories, without the confounding effect of concurrent task practice, is to document experience-driven changes in intrinsic brain connectivity during the post-training resting state, which can be done using a resting state fMRI (rsfMRI) [30]. For instance, Woolley et al. [31] found increased functional connectivity (FC) between hippocampus and caudate nucleus in the resting state after (vs. before) navigation in a virtual water maze, correlated three days later with offline gains in behavioural performance. Likewise, FC increased after 45 min of spatial route learning within a hippocampus-related network [32]. These data indicate that rsfMRI is a sensitive approach to investigate the offline processes subtending the consolidation of novel memories.

From an ecological perspective, topographical learning in unfamiliar environments such as unknown cities may usually span over several days or even weeks, the voyager having the opportunity to visit and revisit different but related spatial units and to integrate the knowledge acquired in previously learned areas with his/her ongoing exploration. In this context, rsfMRI provides a valuable tool to better understand the dynamic processes of integration and consolidation following learning an then relearning, and their relationships with post-training sleep. In the present study, we investigated rsfMRI activity and connectivity changes immediately after spatial navigation learning, at delayed retrieval 4 days later modulated by regular sleep (RS) versus total sleep deprivation (SD) on the first post-learning night, and then after a relearning episode in an extended environment encompassing the initial environment. To do so, we analysed resting-state activity using both seed-based FC (i.e., that estimates the changes in connectivity between predefined seed areas and other brain regions) and amplitude of low frequency fluctuation (ALFF; i.e., that estimates spontaneous activity at rest, regardless of the temporal correlation) metrics. Additionally, task (navigation)-based fMRI activity was recorded during test sessions. We hypothesized that sleep-dependent changes in intrinsic FC in the resting state would parallel and/or complement the previously reported reorganization in task-related brain networks supporting navigation, from hippocampal to striatal and frontal areas [5,23]. Furthermore, we expected further FC changes between the hippocampus, striatum and frontal areas after renewed spatial learning in the extended version of the initial environment, reflecting the ongoing integration with previously consolidated knowledge during sleep.

## 2. Methods

### 2.1. Participants and Procedure

In a first step, 51 healthy volunteers gave their written informed consent to participate in a single experimental session (Day 1) of this study approved by the Ethics Committee of the University of Liege, Belgium (B70720156274). All procedures were performed following relevant guidelines and regulations. One participant was excluded due to an abnormal structural MRI scan. The final sample consisted of 50 right-handed (Laterality Quotient Score [33] > 70) participants (26 males; mean age, 23.5 years; range, 19–32 years). A subset of 34 participants (18 males; mean age, 23.5 years; range, 19–31 years) additionally gave consent to further participate in a SD vs. RS protocol with a second experimental session (Day 4). They were all paid for their participation. Self-reported questionnaires assessed sleep habits (PSQI [34]) and circadian topology (MEQ [35]) for the past month before inclusion. Exclusion criteria were extreme circadian chronotype (MEQ score > 69 or < 31) and bad sleep quality (PSQI score > 5), prior history of neurological or psychiatric disorder, or consumption of psychotropic drugs.

**Figure 1:**
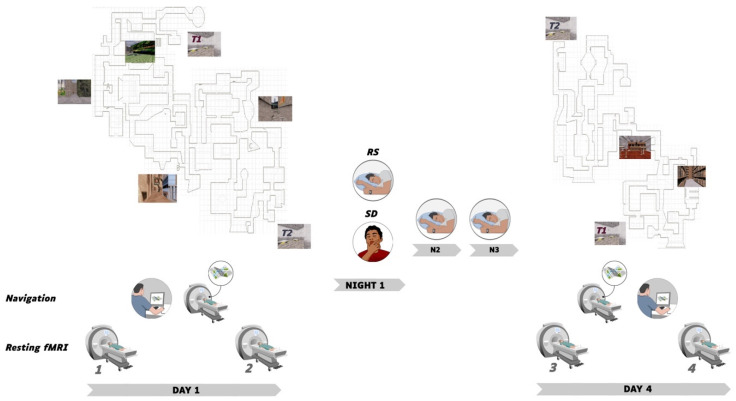
Experimental Protocol. On Day 1, 50 participants were scanned in the resting state before (rsfMRI 1) and after (rsfMRI 2) out-of-scanner exploration of Level 1 of the virtual town, followed by fMRI Test 1 (immediate retrieval navigation test). Thirty-four participants then either slept normally (RS) or were sleep deprived (SD) during Night 1, and then slept normally on Nights 2 and 3. On Day 4, RS and SD participants were scanned in the resting state (rsfMRI 3) followed by fMRI Test 2 (delayed retrieval navigation test). Afterward, they were trained on an extended version (both Level 1 and 2) of the virtual town and then scanned a last time in the resting state (rsfMRI 4). Finally, participants were tested on the extended version of the virtual town outside of the MRI environment (Retrieval extended, not illustrated). Top left and right panels provide an aerial representation of the town map (not seen by participants who navigated from within the environment, see sample pictures) with Level 1 (left, day 1) and 2 (right, day 4) environments and navigation test targets A, B and C located on the 1^st^ level. At Day 4, Level 1 and 2 communicated through teleporters T1 and T2.

The experimental timeline is illustrated in Figure 1. On Day 1, sessions were conducted in the afternoon from 13:00 to 20:00. Participants (*n* = 50) were first scanned during a 5-minute resting state session (rsfMRI 1), then had 45 min outside of the scanner to explore and learn the Level 1 of the virtual city, followed by a behavioural navigation test where they had to reach as fast as possible 10 targets within the learned environment (see below). They were then scanned while performing the navigation test again (i.e., 10 targets to reach) for approximately 8 min, and then scanned again during a second resting state session (rsfMRI 2). A subset of participants was randomly assigned either to the regular sleep (RS; *n* = 16; 9 males) or to the sleep deprivation (SD; *n* = 18; 10 males) condition. Participants in the RS group were allowed normal sleeping at home and were requested to follow regular sleep habits for the 3 experimental nights. In the SD group, participants were kept awake in the laboratory during the first post-learning night. During this SD night, their physical activity was maintained as low as possible under constant supervision by the experimenters. Participants remained most of the time in a seated position, and were allowed to read, chat, play quiet games and watch movies. They received isocaloric meals at regular intervals, and water ad libitum. At 8:00 am, they were allowed to leave the lab, and were instructed to have non-dangerous daytime activities and abstain as much as possible from napping until bedtime. They then slept normally at home the two following nights. Sleep quality and quantity for each night from before the first learning session (Day 1) to the last testing session (Day 4) was assessed using a daily standardized questionnaire [36] controlled by visual inspection of continuous actimetric recordings (ActiGraph, wGT3X-BT Monitor, USA). On Day 4, SD and RS participants came back to the laboratory for a second experimental session. First, they were scanned in the resting state (rsfMRI 3), then scanned using fMRI while engaged in a Level 1 navigation retrieval test similar to Day 1 (i.e., 10 targets to reach). To control for potential circadian confounds, fMRI scanning took place at the same time of day as the initial testing for each individual. Afterwards, participants were trained outside of the MRI scanner for an additional 40 min on an extended version of the navigation task (Level 1 and 2 of the town, see Figure 1), in which were also incorporated the targets and environments of the initial training. They were then scanned again in the resting state (rsfMRI 4) and finally tested on the extended version of the navigation task outside of the MRI environment. A high-resolution structural MRI scan was also acquired for each participant at the end of the brain imaging session, on either Day 1 or Day 4.

### 2.2. Learning Material and Conditions

Participants were trained in a virtual navigation environment previously used in related studies [5,29,37], adapted from a commercially available computer game (Duke Nukem 3D; 3D Realms Entertainment, Apogee Software, Garland, TX, USA) using the editor provided (Build, Ken Silverman, Realms Entertainment, Apogee Software, Garland, TX, USA). It features a complex town made of two levels communicating through two teleports (Figure 1). Each level is composed of three districts, distinct from each other by a different visual architecture, distinctive objects along the streets, and specific background sounds and music. Each district contains one target location identified by a rotating medallion. Participants navigate the town at ground level using a four-direction keypad with their right hand, at a constant speed of 1.25 virtual units per second. 

#### 2.2.1. Behavioural Sessions

During behavioural sessions outside of the scanner (Learning (Day 1) and Relearning (Day 4) phases), participants were trained for about 45 min on the virtual environment presented on a personal computer laptop (screen size, 17 inches). 

On Day 1 (Learning), the training consisted of four 8-minute blocks of free exploration within Level 1 of the virtual environment, each block starting from one of the three possible targets. Participants were explicitly instructed to learn the spatial layout of the streets, districts, and target locations by moving freely within the environment. During the entire training session, pictures of the three target locations and their associated names were continuously available. At the end of each 8-minute blocks, participants were exposed to a test trial similar to the one proposed in the fMRI environment, i.e., they were assigned a starting point and instructed to reach a given target location as fast as possible and in a maximal time of 28 s (i.e., the shortest possible duration from starting point to target destination). At the end of the four 8-minute blocks of free exploration, participants were exposed to 10 test trials in a row, to be performed later in the fMRI setting (see below).

At Day 4 (relearning), participants were trained again for four 8-minute blocks of free exploration within an environment composed of both the initial three districts (Level 1) and an additional level (Level 2) made of three new districts. They were informed that both levels were connected by two teleports positioned at opposite locations and instructed to learn the layout of Level 2 as well as the locations of each teleport, in order to be able to reach the initial targets located on Level 1, starting from random locations in Level 2. Each block started at one of the two teleports. At the end of each 8-minute training block, participants performed a test trial, i.e., they were assigned a starting point in Level 2 and asked to reach, as fast as possible, one of the initial targets in Level 1 within a 60-second window (i.e., the shortest possible duration from Level 2 starting point to Level 1 target destination). After training and the last resting state (rsfMRI 4) sessions, participants were administered a final test of ten 60-second trials outside of the MRI environment. 

#### 2.2.2. Task-Based fMRI

During task-based fMRI (Immediate retrieval fMRI Test 1 and Delayed retrieval fMRI Test 2; Figure 1), volunteers performed a series of navigation tests in the virtual town. For each test, they were assigned a starting point and instructed to reach, as fast as possible, a given target location in Level 1 within the time limit of 28 s. Because the duration of the shortest path between the starting point and the target is 28 s at constant speed, reaching the destination could only be achieved if participants selected the ideal path and did not stop at all during the navigation. The fMRI test session consisted of 10 test blocks, each lasting for 28 s and alternating with a pause menu displayed for a variable duration ranging 10–17 s. For both Day 1 and Day 4 behavioural and fMRI testing sessions, participants’ navigation paths were analysed a posteriori based on video screen recordings, and a quantitative measure of performance was computed at each block as the shortest distance remaining between the subject’s actual location at the end of the time limit for the test trial and the location of the target destination [5,29,37].

#### 2.2.3. Resting State RS-fMRI

During rsfMRI sessions 1, 2, 3 and 4, participants were instructed to relax, keep their eyes open and fixate for 5 min a white cross displayed on a black background on the MRI’s screen. 

### 2.3. Brain Imaging Data Acquisition

Brain MRI data were acquired on a whole-body 3T scanner (Magnetom Prisma, Siemens Medical Solutions, Erlangen, Germany). For both task-based and resting-state fMRI, multi-slice T2*-weighted functional images were acquired with a gradient-echo echo-planar imaging (EPI) sequence using axial slice orientation and covering the whole brain (36 slices, FoV = 216 × 216 mm^2^, voxel size 3 × 3 × 3 mm^3^, 25% interslice gap, matrix size = 72 × 72 × 36, repetition time (TR) = 2260 ms, echo time (TE) = 30 ms, flip angle (FA) = 90 deg). 

For task-based fMRI Retrieval Test 1 and Test 2 sessions, 210 functional images were recorded. The virtual environment was displayed on a screen positioned at the rear of the scanner that the subject could comfortably see through a mirror mounted on the head coil. Participants navigated using 4 directional buttons on a commercially available MRI compatible keypad system (fORP; Current Design, Vancouver). For each resting state session (rsfMRI 1-4), 132 functional images were obtained. 

For anatomical reference, a high-resolution T1-weighted structural image was acquired for each subject (T1-weighted 3D magnetization-prepared rapid gradient echo (MPRAGE) sequence, TR = 1900 ms, TE = 2.19 ms, FA = 9 deg, inversion time (TI) = 900 ms, FoV = 256 × 240 mm^2^, matrix size = 256 × 240 × 224, voxel size = 1 × 1 × 1 mm^3^, acceleration factor in phase-encoding direction R = 2).

### 2.4. Task-Based fMRI Data Analysis

The pre-processing and analysis of functional volumes were performed using the Statistical Parametric Mapping software SPM12 (Wellcome Department of Cognitive Neurology, London, UK) implemented in MATLAB R2012B (Mathworks, Sherbom, MA, USA). The five initial volumes of each time series were discarded to avoid T1 saturation effects. Pre-processing of individual data included realignment (2-step realignment on the first volume of the series), correction for geometric distortions caused by magnetic fields based on the Field Map Toolbox [38], co-registration of functional and anatomical data, spatial normalization into standard stereotactic Montreal Neurological Institute (MNI) space, and spatial smoothing using a Gaussian kernel of 6-mm full width at half maximum (FWHM). Excessive head movements of more than 4 mm of translation or 4 degrees of rotation in any direction were considered exclusion criteria. No participant exhibited excessive head motion. 

Functional data were analysed using a mixed-effects model aimed at showing stereotypical effect in the population from which the subjects are drawn [39]. For each subject, a first-level intra-individual analysis tested effects of interest by linear contrasts convolved with a canonical hemodynamic response function, generating statistical parametric maps. Movement parameters derived from the realignment phase were included as confounding factors. Cut-off period for high-pass filtering was 128 s due to the block design alternating 28-second navigation blocks with 10–17 s breaks. Additionally, navigation performance was added to the model to test possible relationships between neuronal activity in navigation-related areas and behavioural performance. Navigation-related regional BOLD response modulated by navigation performance was computed at the within-subject level. Since no inference was made at this fixed effect level of analysis, individual summary statistic images were computed at a *p* < 0.95 threshold uncorrected, and then further spatially smoothed (6 mm FWHM Gaussian kernel). Individual summary statistics images were then entered in a second-level analysis, corresponding to a random effects (RFX) model, to evaluate commonalities and differences in brain response between the RS and SD groups, and between immediate and delayed Retrieval Test sessions. The resulting set of voxel values for each contrast constituted a map of the *t* statistic (SPM(T)). Statistical inferences were obtained after correction for multiple comparisons at the voxel level (Family Wise Error (FWE) correction *p* < 0.05) in the whole brain, or after small volume correction (SVC) in five regions of interests (ROIs) selected based on published reports showing their involvement in topographical/spatial learning [8,9,40,41,42], i.e., bilaterally the hippocampus, the parahippocampal gyrus, the retrosplenial and medial parietal cortices (Brodmann areas 29, 30 and 31), the dorsal striatum (caudate and putamen) and the entorhinal cortex. Positions and dimensions of ROI were obtained using the Automated Anatomical Labelling (AAL) atlas [43].

### 2.5. Resting State Data Analysis

#### 2.5.1. Preprocessing 

Preprocessing of rsfMRI data was carried out using the SPM12 software and the Data Processing Assistant for Resting-State fMRI (DPARSF) [44]. The first 10 volumes of each time series were discarded to avoid T1 saturation effects. Functional images were realigned to the first volume to correct for head motion. Participants with a translation superior to 2.0 mm in any direction, or an angular motion superior to 2.0 along any axis were excluded from the analysis. Following this criterion, one participant was excluded. The final samples for rsfMRI analyses therefore consisted of 49 participants for day 1, and 34 participants for day 4. Subsequently, each individual structural image (T1-weighted MPRAGE image) was co-registered to the mean functional image after motion correction using a linear transformation. The transformed structural images were then segmented into grey matter (GM), white matter, and cerebrospinal fluid using Diffeormorphic Anatomical Registration Through Exponentiated Lie Algebra (DARTEL). To further reduce potential confounds of head motion, a Friston-24 correction [45] was applied based on six rigid body head motion parameters. Signals from cerebrospinal fluid and white matter were also entered as confound covariates to reduce the possible effects of physiological artifacts. The corrected functional volumes were spatially normalized to the Montreal Neurological Institute (MNI) space and re-sampled to 3 mm isotropic voxels using DARTEL. Resultant normalized functional images were de-trended and spatially smoothed with an 8 mm full-width-at-half-maximum (FWHM) Gaussian kernel. 

#### 2.5.2. ALFF Analysis

Smoothed images were band-pass filtered (0.1–0.01 Hz) to reduce low-frequency drifts and high-frequency noise before the Amplitude of Low Frequency Fluctuations (ALFF) analysis aimed at investigating local changes in resting state activity. ALFF individual maps were computed with DPARSF [44] and entered into second-level analyses to examine increases in ALFF signal associated with a spatial learning episode on Day 1 and Day 4, and to investigate if functional changes associated with learning in the extended version of the environment differed between SD and RS groups on Day 4. ALFF changes associated with performance were also investigated by entering behavioural performance as a covariate of interest at the second level (RFX) of the analysis. Statistical inferences were computed at the whole brain level based on a two-tailed permutation test with 5000 permutations and a threshold-free cluster enhancement (TFCE) correction within a grey matter mask (obtained by thresholding an a priori grey matter probability map in SPM12; threshold = 0.25). Results were considered significant at *p* < 0.05 after a false discovery rate (FDR) correction for multiple comparisons. Region of interest analyses were also conducted within the five a priori ROIs defined above based on the literature [8,9,40,41,42]. Clusters in which activations were found to correlate with performance in the task-based fMRI analysis on Day 1 and/or Day 4 were also examined as regions of interests for Day 1 (3 clusters) and Day 4 (0 clusters) ALFF analyses.

#### 2.5.3. Seed-Based Functional Connectivity Analysis

FC was analysed with seed-voxel correlation mapping using the CONN-fMRI toolbox 13.i for SPM [46]. Preprocessed data (see above) were temporally filtered using a band-pass filter to retain frequencies from 0.008 to 0.09 Hz. Noise signals such as cerebrospinal fluid, white matter, movement parameters and time-series predictors of global signal were further removed from the images using the component-based noise correction method (CompCor) [47]. Averaged signals were extracted from 10 different seeds defined using the Automated Anatomical Labelling (AAL) atlas: left and right hippocampus, parahippocampus, dorsal striatum, retrosplenial-medio parietal cortex and entorhinal cortex (regions of interest taken from [8,9,40,41,42]). Clusters found to correlate with performance in the task-based fMRI and ALFF analyses on Day 1 and Day 4 were also used as seeds for Day 1 and Day 4 analyses respectively, yielding 7 extra seeds for Day 1 and 2 extra seeds for Day 4. Temporal correlations of resting-state BOLD signal time series between these seeds and the rest of the brain were examined using a General Linear Model approach. Individual connectivity maps were computed for each seed and entered into a second level analysis to examine changes in connectivity following navigation learning on Day 1, and to investigate if functional changes associated with learning in the extended version of the environment differed between RS and SD groups on Day 4. Behavioural performance following learning was used as a covariate of interest (or as a weighting factor of BOLD signal). Statistical inference for each seed and contrast were first based on a family-wise error correction (*p* < 0.005) at the cluster-level with an uncorrected threshold of *p* < 0.001 at the voxel level. A Bonferroni correction was then applied based on the number of seeds for Day 1 (*n* = 17) and Day 4 (*n* = 12) analyses separately. Results were thus considered significant at a threshold of *p* = 0.0014 (.05/17) for Day 1 analyses and *p* = 0.002 (.05/12) for Day 4 analyses. 

## 3. Results

### 3.1. Behavioural Results

#### 3.1.1. Sleep Data 

Sleep duration and quality were estimated by means of self-reports of the nights preceding Day 1 and Day 4. Mean sleep duration was not significantly different between RS and SD groups on the night before Day 1 (RS = 437 ± 60 min, TSD = 457 ± 46 min; *p* > 0.2) and the night before Day 4 (RS = 436 ± 55 min, TSD = 459 ± 48 min; *p* > 0.2). Within each group, duration of each night did not differ significantly (RS group: *p* > 0.8; TSD group: *p* > 0.7). Likewise, a similar analysis conducted on subjective sleep quality assessed by means of a 6-point scale failed to evidence between-group differences. Subjective sleep quality was equivalent between groups for each night (night before Day 1: RS = 3.7 ± 1.2, TSD = 3.4 ± 1.1; *p* > 0.5; night before Day 4: RS = 3.7 ± 0.7, TSD = 3.7 ± 0.9; *p* > 0.9). Finally, within-group comparisons did not reveal any significant difference in sleep quality between both nights (RS group: *p* > 0.9; TSD group: *p* > 0.1). These results indicate that sleep patterns observed the night before the SD episode were restored before being scanned again in the delayed session, and therefore that all participants were tested under similar states of alertness on Days 1 and 4.

#### 3.1.2. Navigation Performance

As a reminder, for each of the 10 place finding tests administered during fMRI sessions at Day 1 (Immediate retrieval) and Day 4 (Delayed retrieval), participants were instructed to reach, as fast as possible, a given Level 1 target from one designated starting point within 28 s. Distance remaining to reach destination was the estimate of navigation performance [4,5,29] (see Methods). Mean distance scores per session for the SD group were 17.17 (arbitrary units, standard deviation (sd 8.29)) and 17.53 (sd 11.1) at immediate (Day 1) and delayed retrieval (Day 4), respectively. Distances scores for the RS group were 15.12 (sd 13.15) at immediate and 15.47 (sd 12.2) at delayed retrieval, respectively. A two-way ANOVA computed on within-session individual mean scores with group (SD vs. RS) and retrieval session (Immediate vs. Delayed) factors did not reveal any significant main or interaction effect (all *ps* > 0.1). Likewise, at testing after the second learning session on the extended version of the town, mean performance did not statistically differ (*t* = −0.2; *p* = 0.8) between the RS (arbitrary units, 15.8, sd 19.10) and SD (17.3, sd 19.7) conditions. 

These results suggest that (1) both groups gained knowledge of the virtual town that persisted 3 days after learning, that (2) SD on the first post-training night did not overtly alter subjects’ ability to find their way in the town at delayed retrieval (which does not preclude the use of qualitatively different strategies for a similar performance), and that (3) learning novel routes and integration with prior knowledge in the extended version of the town was similar in the RS and SD conditions.

### 3.2. Task-Based fMRI

#### 3.2.1. Navigation-Related Brain Activity 

A conjunction analysis disclosed increased blood–oxygen level-dependent (BOLD) responses in an extended hippocampo-neocortical network during place finding, both at immediate (Day 1) and delayed (Day 4) navigation retrieval sessions. At immediate retrieval, navigation-related activity was found mostly in the right hippocampus/parahippocampus (32 −40–4 mm in MNI standard stereotactic space, Z score = 4.55, number of voxels in cluster k = 30; p^svc^ < 0.05) and surrounding structures as well as in occipital, parietal, frontal and cerebellar areas (Supplementary Table S1). At delayed retrieval, increased BOLD activity was highlighted in a similar set of brain structures, except for the right hippocampus, in which activation did not survive correction for multiple comparisons (Supplementary Table S2). Correlation analyses disclosed a positive relationship between individual performance scores (i.e., reversed navigation score based on the distance toward destination, higher scores meaning better performance) and navigation-related responses in the left (−22 2–10 mm, Z = 4.14, k = 74, p^svc^ < 0.03) putamen at immediate retrieval. At delayed retrieval, no significant correlation was found between performance and navigation-related activity, either at the whole brain level or within ROIs. 

#### 3.2.2. Post-Training Sleep-Dependent Reorganization of Navigation-Related Brain Activity

A group (SD vs. RS) by session (Day 1 vs. Day 4) ANOVA conducted on navigation-related brain activity did not disclose any main or interaction effect, either at the whole brain level or within ROIs. A similar analysis conducted on navigation-related regional BOLD responses modulated by navigation performance also did not yield significant results.

### 3.3. Resting State ALFF

#### 3.3.1. Spatial Learning-Related Changes in Spontaneous Activity at Rest

On Day 1, local ALFF increased following learning (rsfMRI 2 vs. rsfMRI 1) in a parietal medial cluster encompassing parts of the precuneus and of the posterior cingulate gyrus (peak x y z coordinate 6 –45 21 mm, intensity = 479.36, cluster extent k = 268) and an extended cluster encompassing frontal and parietal regions (6 −30 60 mm, intensity = 513.80, k = 409; all *ps^FDR^* < 0.05). On Day 4, local ALFF increased following learning (rsfMRI 4 vs. rsfMRI 3) in a parietal cluster (maximal peak −3 –57 15 mm, intensity = 788.80, k = 4072), a cerebellar cluster (−36 −60 −42 mm, intensity = 528.01, k = 130) and two clusters located in the left rectal gyrus (0 24 –21 mm, intensity = 623.98, k = 271) and the right lingual gyrus (15 −99 −12 mm, intensity = 593.63, k = 463; all *ps^FDR^* < 0.05).

On Day 1, there was a correlation between navigation retrieval performance (Test 1) and increased ALFF in the right and left posterior cingulate (3 –51 27 mm, intensity 399.51, k = 74; −3 −54 27 mm, intensity = 104.69, k = 57) and right parahippocampal gyrus (33 −18 −30 mm, intensity 50.38, k = 24; all *ps^svc^* < 0.05) clusters. On Day 4, navigation performance correlated with local ALFF in left parahippocampal gyrus (−15 −6 −15 mm, intensity = 72.10, k = 24; −24 −6 −33 mm, intensity = 95.99, k = 61; all *ps^svc^* < 0.05) clusters. 

#### 3.3.2. Post-Training Sleep-Dependent Learning-Related Changes in Spontaneous Activity at Rest

Between-group comparison revealed no significant differences in ALFF changes associated with learning on either Day 1 or Day 4 when comparing RS and SD groups. 

### 3.4. Resting State FC

#### 3.4.1. Spatial Learning-Related Changes in Seed-Based FC 

As a reminder, seeds for connectivity analyses were defined based on a priori ROIs (10 seeds) and on clusters found to correlate with performance in the task-based fMRI or ALFF analyses (see Methods section) for a total of 16 seeds for Day 1 and 12 seeds for Day 4. All analyses are Bonferroni corrected for multiple comparisons. On Day 1 (corrected significance threshold *p* < 0.05/16 = 0.0031), increased FC (from pre-learning (rsfMRI 1) to post-learning (rsfMRI 2) resting state sessions) was found between the left hippocampus and the right middle frontal gyrus (32 4 56 mm, k = 247, T = 4.95, cluster p^FWE^ = 0.0023), between the right entorhinal cortex and three clusters located in the right temporal (52 −18 −24 mm, k = 351, T = 5.46, cluster p^FWE^ = 0.000001) and left temporal (−58 −16 −24 mm, k = 401, T = 5.02, cluster p^FWE^ = 0.000066) cortices, and in the right posterior cingulum (10 −42 28 mm, k = 605, T = 4.18, cluster p^FWE^ = 0.000197), as well as between the left entorhinal cortex and a cluster located in the cerebellum (−30 −40 −34 mm, k = 310, T = 4.88, cluster p^FWE^ = 0.000497). Based on seeds taken from task-based correlation analyses between navigation-related activity and performance, spatial learning on Day 1 was associated with increased FC between the left parahippocampal gyrus and the right precuneus (6 −60 44 mm, k = 330, T = 5.09, cluster p^FWE^ = 0.000211), the right precuneus and the right middle frontal gyrus (30 4 38 mm, k = 324, T = 4.26, cluster p^FWE^ = 0.000343) and the left posterior cingulate cortex and the right supramarginal gyrus (58 −34 48 mm, k = 232, T = 4.57, cluster p^FWE^ = 0.000377; see Table 1 for detailed results). No FC change correlated with performance. 

On Day 4 (corrected significance threshold *p* < 0.05/12 = 0.0041), learning in the extended version of the task triggered increased FC (from the pre-learning (rsfMRI 3) to the post-learning (rsfMRI 4) resting state session) between the left hippocampus and the right postcentral gyrus (36 −30 70 mm, k = 249, T = 4.34, cluster p^FWE^ = 0.002026). FC change did not correlate with performance. 

#### 3.4.2. Sleep-Dependent Changes in Seed-Based Resting State FC 

**Figure 2:**
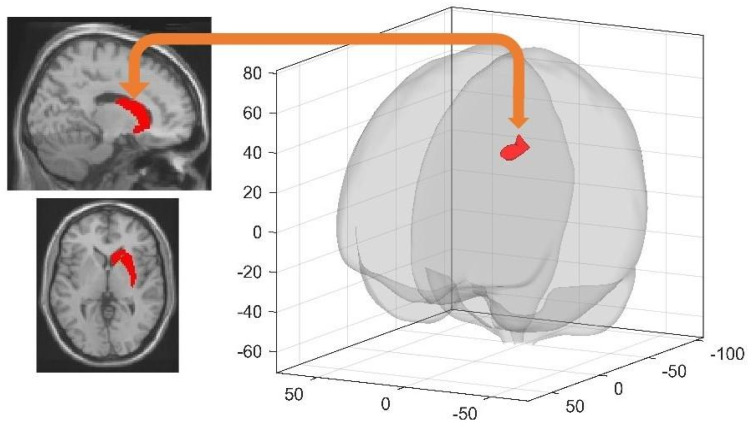
Higher FC in RS than SD participants between the right dorsal striatum (seed; left panel) and the middle frontal gyrus (target; right panel; cluster p^FWE^ = 0.001851) at Day 4 prior to relearning. Target clusters are visualised using the CONN toolbox glass display [46]. Seed areas are displayed on the mean MNI template (SPM12 software, Wellcome Department of Cognitive Neurology, London).

At Day 4 during the first resting state session (rsfMRI 3) before extended learning in the maze, RS participants exhibited higher FC than SD participants between the right dorsal striatum and the left middle frontal gyrus (−34 30 42 mm, k = 272, T = 4.71, cluster p^FWE^ = 0.001851) (Figure 2). As a proof of concept, we ran similar between-group (RS vs. SD) comparisons on pre- (rsfMRI 1) and post- (rsfMRI 2) learning resting state sessions on Day 1 to ensure that sleep-related differences observed in rsfMRI 3 at Day 4 cannot be explained by initial between-group differences. No significant FC differences between RS and SD conditions were found for any of the 16 seeds considered for the FC analysis on Day 1. Similarly, no significant between-group differences were found when considering learning-dependent changes in FC (rsfMRI 2 > rsfMRI 1). Altogether, it suggests that between-group differences observed at the Day 4 rsfMRI 3 session are attributable to the sleep manipulation on the first post-training night.

#### 3.4.3. Post-Training Sleep-Dependent Relearning-Related Changes in Seed-Based Resting State FC 

**Figure 3:**
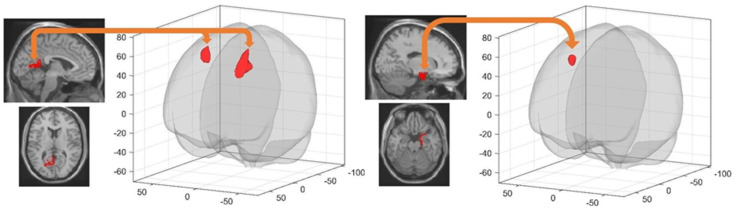
Higher seed-based FC in SD than RS participants between (left panel) left retrosplenial cortex (seed) and two frontal clusters (target left superior frontal gyrus and right middle frontal gyrus; cluster p^FWE^ > 0.00005) and (right panel) right entorhinal cortex (seed) and a right frontal gyrus cluster (target; cluster p^FWE^ < 0.002). Target clusters are visualised using the CONN toolbox glass display [46]. Seed areas are displayed on the mean MNI template (SPM12 software, Wellcome Department of Cognitive Neurology, London).

A Group (RS vs. SD) by resting state Session (pre-extended learning (rsfMRI 3) vs. post-extended learning (rsfMRI 4)) interaction analysis investigated sleep-dependent changes in resting state FC after learning on the extended version of the town at Day 4. This analysis disclosed increased FC from pre- to post-learning in the SD as compared to the RS condition, between the left retrosplenial cortex and the left superior frontal gyrus (−16 12 48 mm, k = 738, T = 5.91, cluster p^FWE^ < 0.000001), between the left retrosplenial cortex and the right middle frontal gyrus (28 6 56 mm, k = 390, T = 5.78, cluster p^FWE^ = 0.000047), and between the right entorhinal cortex and the right superior frontal gyrus (18 24 56 mm, k = 228, T = 5.25, cluster p^FWE^ = 0.001829; Table 2). A post-hoc analysis investigating the directionality of the interaction effects highlighted significantly decreased FC in the RS condition vs. increased FC in the SD condition between the left retrosplenial cortex and the left superior frontal gyrus (RS, β_pre-learning_ = 0.28_,_ β_post-learning_ = 0.05, T = 4.44, *p* < 0.001; SD, β_pre-learning_ = 0.12_,_ β_post-learning_ = 0.26, T = 3.13, *p* < 0.001), the left retrosplenial cortex and the right middle frontal gyrus (RS, β_pre-learning_ = 0.13_,_ β_post-learning_ = −0.07,T = 4.38, *p* < 0.001; SD, β_pre-learning_ = −0.08_,_ β_post-learning =_ 0.01, T = 2.94, *p* = 0.004) and the right entorhinal cortex and the right frontal superior gyrus (RS, β_pre-learning_ = 0.07_,_ β_post-learning_ = −0.09, T = 5.19, *p* < 0.001; SD, β_pre-learning_ = −0.15_,_ β_post-learning_ = −0.02, T = 2.80, *p* = 0.006) (Figure 3).

## 4. Discussion

In the present study, resting-state fMRI was used to investigate the offline changes in brain functional connectivity (seed-based FC) and intrinsic regional activity (ALFF) potentially associated with learning and relearning following sleep in a spatial navigation task.

In line with previous studies, behavioural performance was similar between groups sleep deprived (SD) or sleep rested (RS) after learning, not only at immediate but also at delayed retrieval [5,23], or when tested after learning the extended version of the town. It suggests that one night of SD following training is insufficient to induce observable changes at the behavioural level (see [5,48]), which does not exclude the development of distinct neural strategies subtending successful navigation [5,11,23]. Indeed, prior studies found a shift between neural structures subtending quantitatively equivalent retrieval performance after sleep on the post-training night, from hippocampus to striatum in the virtual navigation task after a few days [5,23], and from hippocampus to neocortical frontal regions in other declarative memory tasks after a sleep episode in children [24] and after weeks to months in adults [49]. Noticeably, we avoided testing participants at the outcome of the SD experimental night as the stress of the SD experience (and fatigue) would have impacted both performance and brain activity in a way that would actually mask offline sleep-dependent memory consolidation effects. Allowing two regular sleep nights to all participants after the experimental night ensured that the observed differences in brain activity were not attributable to being in an SD state at the time of scanning. Moreover, although it cannot be excluded that some participants took a daytime nap after the SD night, this does do not undermine our findings because we investigated here the effect of the first post-training night of nocturnal sleep, and naps in this case represent a much-delayed period of sleep (just as were the 2 recovery nights), which was, moreover, obtained at a suboptimal circadian phase, as previously discussed [5].

Learning to navigate in the virtual environment on Day 1 was associated with increased FC between the left hippocampus and the right middle frontal gyrus, as well as in a network comprising the right entorhinal cortex, bilateral temporal regions and the right posterior cingulum cortex. The right middle frontal gyrus is active during virtual maze exploration [50], and is important for episodic memory retrieval [51], while the cingulum contributes to the sense of self-location [52] and the entorhinal cortex plays a key role for the encoding of map-like spatial codes [53]. Consequently, increased FC may subtend the integration of egocentric and allocentric spatial representations. When considering as seeds the clusters obtained from task-related fMRI and ALFF analyses, increased FC was found between the left parahippocampal gyrus and the right middle frontal gyrus, a set of regions centrally related to spatial memory [54,55,56]. On Day 4, learning the extended version of the town triggered an increase in FC between the left hippocampus and the right postcentral gyrus, two regions subtending spatial navigation in recently learned environments [57]. Noticeably, recent reports propose that, besides being a somatosensory area, the postcentral gyrus plays a role as a high-order navigational system. In rats, spatial cells in this system elicit similar firing characteristics than the ones in the hippocampus [58]. To the best of our knowledge, this finding has not yet been replicated in humans. Notwithstanding, task-dependent increased FC between the hippocampus and somatosensory areas may be seen as evidence supporting the hypothesis of a cooperation between these two navigational systems. 

Sleep deprivation effects on resting-state activity were tested as soon as participants arrived at the laboratory (Day 4), before any kind of learning-related stimulus was presented. Interestingly, we found higher FC in RS than SD participants between the right dorsal striatum and the left middle frontal gyrus. Although supporting behavioural evidence is lacking, it indicates a sleep-dependent reorganization of brain functioning in areas recruited during learning and navigation.

Learning to navigate in an extended version of the initial environment was associated with increased FC between navigation-related brain structures (left retrosplenial and right entorhinal cortices) and frontal regions (left and right superior frontal and right middle frontal gyri) in participants deprived of sleep the night after initial learning (SD) as compared to those having slept normally (RS). The entorhinal and the frontal cortices are respectively thought to support landmark recognition and strategy planning [59] in the context of spatial navigation, while the retrosplenial cortex plays a central role in spatial strategy switching [60,61]. Stronger functional connections with frontal regions in SD participants might reflect the compensatory brain activity needed to develop different but quantitatively equivalent (eventually leading to similar behavioural performance) spatial representation strategies in the context of extended learning in a less strongly consolidated environment. This tentative interpretation should be tested in further studies. 

We also investigated changes in the amplitude of low frequency fluctuation (ALFF) as potential markers of learning and memory consolidation processes [62,63]. Both on Day 1 and Day 4, pre- to post-(re)learning changes in local brain activity (ALFF) at rest partially overlapped with clusters found active when performing on the navigation task, suggesting the offline continuation of task-related activity during post-training wakefulness [29]. Amongst the regions found active in both online (task) and offline (resting state) post-training conditions in the present study, the precuneus was previously involved in self-related mental representations both during rest and spatial information processing [64]. Post-training activations in the precuneus may thus support the creation of the egocentric spatial map needed for successful topographical navigation. Increased spontaneous activity (ALFF) after learning was also detected on Day 1 in the posterior cingulum, a region that plays an important role in visual-spatial memory due to its connections with hippocampal and parahippocampal regions [65]. At Day 4, ALFF increased in the right lingual gyrus, a part of the parahippocampal place area (PPA) that responds preferentially to complex visual scenes such as landscapes or cityscapes [66]. However, the lack of specific literature relating ALFF with memory consolidation processes calls for caution in the interpretation; it might be premature at this stage to link ALFF changes to the replay of memory traces. Both on Day 1 and Day 4, increased resting state activity in the medial temporal lobe positively correlated with behavioural navigation performance, suggesting that prior learning modulates activity in this brain region that to some extent reflects successful learning. In line with behavioural and task-fMRI results, the group (SD vs. RS) by session interaction on spontaneous resting state activity was non-significant, suggesting that ALFF measurements mostly reflected immediate post-training memory processes in waking, but were not modulated by SD after the first post-training night.

At both immediate and delayed retrieval, task-based fMRI analyses evidenced navigation-related brain activity in a large network including frontal and occipital areas, similar to the ones previously found to support navigation in this task [5,37]. In the current study, however, at variance with previous studies from our group reporting significant correlations between performance and medial temporal activity [5,23] at immediate retrieval, performance at Day 1 already correlated with striatal activation. Consequently, we could not replicate the observation of a shift from hippocampal to a caudate-dependent spatial strategy in the RS as compared to the SD group [5,23], striatal activation being already present at Day 1. We propose here three possible explanations for this discrepancy. First, it should be taken into account that participants spent approximately 20 min lying in the MRI scanner prior to the training session, which might have augmented stress levels and sleepiness. In animals, these elements have been proposed to lead to an increased use of the striatal spatial memory system [67]. Second, at variance with our prior study [5], the behavioural training proposed to participants on Day 1 included at the end a repetition of the 10 tests administered during the subsequent task-based fMRI session. This final test was added to reinforce the memory traces and habituate our participants to the conditions in which they would have to perform next in the scanner. However, as participants experienced already once navigating in these conditions, this may have eventually resulted (and much faster than we expected) in a form of automation of the navigation, with a shift toward a striatum-based strategy. Third, the results obtained in our prior study [5] now date back more than 12 years. Since that time, the use of geolocation software to navigate in the real world has become a given for the new generations, and especially for young adult volunteers. Thus, it cannot be excluded that the spontaneous neuronal underpinnings of spatial navigation performance have evolved in unexpected ways due to the quasi-constant availability of external navigation systems that to some extent replace the need to create topographical representations, a hypothesis that needs experimental confirmation. Finally, the study protocol (as the previous ones [5,23]) does not include a control condition in which participants would have trained on an exploration experience not implicating learning, which would have given us a more specific aspects of learning dynamics. Similarly, the results regarding the interaction between post-learning sleep deprivation and the extension of a previously studied virtual environment would require further confirmation in a larger cohort by using a control condition in which half of the participants of each group (RS and SD) are trained on a completely unrelated environment on Day 4.

## 5. Conclusions

Altogether, our results highlight the continuation of navigation-related activity in the subsequent resting state, as evidenced by changes in functional connectivity and the amplitude of low frequency fluctuations in task-related neural networks. Furthermore, sleep deprivation on the post-training night was associated with increased FC between navigation-related brain structures when faced to the task to learn a novel but related environment (i.e., an extended version of the town), suggesting that SD triggered the need to recruit more resources to link novel information with possibly less efficiently consolidated (after SD) existing memory traces. 

## Figures and Tables

**Table 1:** Spatial learning related changes in resting state FC on Day 1 (rsfMRI 2 > rsfMRI 1).

Seed Area	Target Brain Region(s)	x y z (mm)	K	T	Cluster p^FWE^ (Threshold < 0.0029)
Left Parahippocampal Gyrus *	Right Precuneus	6 −60 44	330	5.09	0.000211
Right Precuneus *	Right Middle Frontal Gyrus	30 4 38	324	4.26	0.000343
Cingulum_Post_L *	Right Supramarginal Gyrus	58 −34 48	232	4.57	0.000377
Left Hippocampus	Right Middle Frontal Gyrus	32 4 56	247	4.95	0.002300
Right Entorhinal Cortex	Right Inferiotemporal Gyrus	52 −18 −24	351	5.46	0.000001
	Left Middle Temporal Gyrus	−58 −16 −24	401	5.02	0.000066
	Right Post Cingulate Gyrus	10 −42 28	605	4.18	0.000197
Left Entorhinal Cortex	Left lobule VI of cerebellar hemisphere	−30 −40 −34	310	4.88	0.000497

Note. MNI coordinates (mm) indicate peak-voxel location. FEW = Family Wise Error. * seeds obtained from task-related fMRI or ALFF analysis. K = cluster size expressed in number of voxels.

**Table 2:** Post training Sleep-Dependent Relearning-Related Changes in resting state seed-based FC on Day 4 (rsfMRI 4 > rsfMRI 3, SD > RS).

Seed Area	Target Brain Region(s)	x y z (mm)	k	T	Cluster p-FWE
Left Retrosplenial Cortex	Left Superior Frontal Gyrus	−16 12 48	738	5.91	0.000000
	Right Middle Frontal Gyrus	28 6 56	390	5.78	0.000047
Right Entorhinal Cortex	Right Superior Frontal Gyrus	18 24 56	228	5.25	0.001829

Note. MNI coordinates indicate peak-voxels location. FEW = Family Wise Error. K = cluster size expressed in number of voxels.

## Data Availability

The data presented in this study are available on request from the corresponding author. The data are not yet publicly available due to scientific agreement between parties who process related elements in the datasets.

## Supplementary Materials

The following are available online at https://www.mdpi.com/article/10.3390/brainsci11040476/s1, Table S1: Clusters of Significant Increased Blood–oxygen level-dependent (BOLD) Responses during Spatial Navigation at Immediate retrieval (Day 1), Table S2: Clusters of Significant Increased Blood–oxygen level-dependent (BOLD) Responses during Spatial Navigation at Delayed retrieval (Day 4).
