# Supplementary material for: Post-Training Sleep Modulates Topographical Relearning-Dependent Resting State Activity
Source: Brain Sci. 2021 Apr 9;11(4):476. doi: 10.3390/brainsci11040476 (PMC8069225; doi:10.3390/brainsci11040476)
Supplement: Supplementary file 1 [file brainsci-11-00476-s001.pdf]

Post-training sleep modulates topographical relearning-dependent resting state activity

Michele Deantoni, Thomas Villemonteix, Evelyne Balteau, Christina Schmidt and Philippe Peigneux

SUPPLEMENTARY INFORMATION

Table S1. Clusters of Significant Increased Blood–oxygen level-dependent (BOLD) Responses during Spatial Navigation at Immediate retrieval (Day 1)

**Brain area**	**Peak-coordinate (MNI)** x y z (mm)	**Cluster size**	**p-value** (FWE-corr)
Inferior Semi-Lunar Lobule (Cerebellum)	0 -60 -36	3919	<.001
Right Superior Occipital	34 -82 26	2674	<.001
Left Middle Frontal Gyrus	-20 -2 62	1619	<.001
Right Middle Frontal Gyrus	24 2 58	584	<.001
Left Parahippocampal Gyrus	-22 -44 -8	518	<.001
Left Cuneus	-24 -82 36	1624	<.001
Left Posterior Cingulate	-18 -58 14	250	<.001
Left Cerebellar Tonsil	-16 -56 -42	92	<.001
Right caudate tail	22 -30 14	30	<.001
Left Cingulate Gyrus	-6 6 46	165	<.001
Left Cingulate Gyrus	-12 -18 44	59	0.001
Thalamus (Pulvinar)	-16 -26 16	18	0.003
Left Claustrum	-28 24 4	22	0.005
Thalamus	-6 -28 -6	11	0.006
Dorsal Striatum	-22 2 -10	74	.027 **

All values FWE = Family-Wise Error corrected at whole brain level, excepted ** after small volume correction in ROI; MNI = Montreal Neurological Institute. Only clusters with a size > 10 voxels are reported.

Table S2. Clusters of Significant Increased Blood–oxygen level-dependent (BOLD) Responses during Spatial Navigation at Delayed retrieval (Day 4)

**Brain area**	**MNI space** x y z (mm)		**p-value** (FWE-corr)
Inferior Semi-Lunar Lobule (Cerebellum)	0 -64 -38	17692	<.001
Thalamus	-6 -28 -2	587	<.001
Right Insula	36 18 6	239	<.001
Lentiform Nucleus (Putamen)	-24 4 12	280	<.001
Right Pulvinar (Thalamus)	20 -26 14	72	<.001
Right Cingulate Gyrus	14 -18 44	148	<.001
Right Fusiform Gyrus	50 -60 -8	36	<.001
Right Middle Frontal Gyrus	32 38 32	50	0.004
Precentral Gyrus	-44 0 8	14	0.008
Superior Temporal Gyrus	-42 -32 16	23	0.009
Left Dorsal Striatum	-24 4 12	542	<.001 **
Right Dorsal Striatum	24 20 0	452	<.001 **
Right Hippocampus	18 -32 -4	5	.041 **
Left Parahippocampal Gyrus	-16 -56 10	843	<.001 **
Right Parahippocampal Gyrus	30 -44 -6	23	<.001 **
Left Retrosplenial Cortex	-16 -58 10	226	<.001 **
Right Retrosplenial Cortex	4 -74 4	249	<.001 **

All values FWE = Family-Wise Error corrected at whole brain level, excepted ** after small volume correction in ROI; MNI = Montreal Neurological Institute. Only clusters with a size > 10 voxels are reported.
